# *Atm *heterozygous deficiency enhances development of mammary carcinomas in *p53 *heterozygous knockout mice

**DOI:** 10.1186/bcr968

**Published:** 2004-12-10

**Authors:** Seiichi Umesako, Kae Fujisawa, Sayoko Iiga, Nobuko Mori, Masahiro Takahashi, Doo-Pyo Hong, Chang-Woo Song, Satomi Haga, Syunsuke Imai, Otsura Niwa, Masaaki Okumoto

**Affiliations:** 1 Graduate school of Agriculture and Biological Sciences, Osaka Prefecture University, Osaka, Japan; 2 Research Institute of New Medicines, Shionogi Pharmaceutical Co., Osaka, Japan; 3 Research Institute for Advanced Science and Technology, Osaka Prefecture University, Osaka, Japan; 4 Korea Research Institute of Chemical Technology, Taejon, Korea; 5 Department of Anatomy, Nara Medical University, Nara, Japan; 6 Nara Prefecture Institute for Hygiene and Environment, Nara, Japan; 7 Radiation Biology Center, Kyoto University, Kyoto, Japan

**Keywords:** *Atm*, mammary carcinoma, mouse, *p53*, radiation

## Abstract

**Introduction:**

Ataxia-telangiectasia is an autosomal-recessive disease that affects neuro-immunological functions, associated with increased susceptibility to malignancy, chromosomal instability and hypersensitivity to ionizing radiation. Although ataxia-telangiectasia mutated (*ATM*) heterozygous deficiency has been proposed to increase susceptibility to breast cancer, some studies have not found excess risk. In experimental animals, increased susceptibility to breast cancer is not observed in the *Atm *heterozygous deficient mice (*Atm*^+/-^) carrying a knockout null allele. In order to determine the effect of *Atm *heterozygous deficiency on mammary tumourigenesis, we generated a series of *Atm*^+/- ^mice on the *p53*^+/- ^background with a certain predisposition to spontaneous development of mammary carcinomas, and we examined the development of the tumours after X-irradiation.

**Methods:**

BALB/cHeA-*p53*^+/- ^mice were crossed with MSM/Ms-*Atm*^+/- ^mice, and females of the F_1 _progeny ([BALB/cHeA × MSM/Ms]F_1_) with four genotypes were used in the experiments. The mice were exposed to X-rays (5 Gy; 0.5 Gy/min) at age 5 weeks.

**Results:**

We tested the effect of haploinsufficiency of the *Atm *gene on mammary tumourigenesis after X-irradiation in the *p53*^+/- ^mice of the BALB/cHeA × MSM/Ms background. The singly heterozygous *p53*^+/- ^mice subjected to X-irradiation developed mammary carcinomas at around 25 weeks of age, and the final incidence of mammary carcinomas at 39 weeks was 31% (19 out of 61). The introduction of the heterozygous *Atm *knockout alleles into the background of the *p53*^+/- ^genotype significantly increased the incidence of mammary carcinoma to 58% (32 out of 55) and increased the average number of mammary carcinomas per mouse. However, introduction of *Atm *alleles did not change the latency of development of mammary carcinoma.

**Conclusion:**

Our results indicate a strong enhancement in mammary carcinogenesis by *Atm *heterozygous deficiency in *p53*^+/- ^mice. Thus, doubly heterozygous mice represent a useful model system with which to analyze the interaction of heterozygous genotypes for *p53*, *Atm *and other genes, and their effects on mammary carcinogenesis.

## Introduction

Ataxia-telangiectasia is an autosomal-recessive disease that affects neuro-immunologic functions, and is associated with increased susceptibility to malignancy, chromosomal instability and hypersensitivity to ionizing radiation [1,2]. *ATM *(ataxia-telangiectasia mutated) heterozygous deficiency has been proposed to increase susceptibility to breast cancer [3-7]. However, those early studies were limited by the lack of reliable assays with which to identify carriers [8]. In fact, in a later study a lack of association of heterozygous *ATM *mutations with early onset of breast cancer was found [9]. More recent epidemiological studies suggested that missense mutation in the *ATM *gene, rather than a protein-truncating mutation, which accounts for the majority of mutations in patients with ataxia-telangiectasia, confers increased risk for breast cancer [10]. Thus, cancer risk in *ATM *heterozygotes varies depending on the mutation type (i.e. some missense-type mutations are associated with early onset of breast carcinoma whereas truncation-type mutations are not) [11,12]. Recently, epidemiological studies on excess risk for breast cancer in *ATM *heterozygosity were reported [13].

In experimental animals, no tumours were observed in *Atm *heterozygous mice carrying a knockout null allele of *Atm *[14]. In contrast, *Atm *knock-in heterozygous mice harbouring an in-frame deletion corresponding to the human mutation exhibit increased susceptibility to a wide variety of tumours [14]. Thus, data in humans and mice suggest that the type of *Atm *mutation determines susceptibility to cancer in heterozygous individuals. Heterozygosity for a null knockout allele of *Atm *in mice and protein-truncating alleles of *ATM *in humans was thought not to increase susceptibility to mammary cancer. On the other hand, haploinsufficiency at the *Atm *gene has a phenotype of increased sensitivity to ionizing radiation in mice [15].

In contrast to the *Atm *gene, the *p53 *null allele exhibits haploinsufficiency for the development of tumours in mice, mainly lympho-haematopoietic malignancies [16,17]. The *p53 *heterozygotes of BALB/c genetic background develop mammary tumours [18-20]. Mice doubly null for the *p53 *and *Atm *genes were reported to exhibit a dramatic acceleration in tumour formation relative to singly null mice, indicating that the genes cooperate in a significant manner to prevent tumourigenesis [21]. However, the authors noted no mammary carcinoma in any of the four genotypes studied (*p53*^+/+ ^*Atm*^-/-^, *p53*^+/- ^*Atm*^-/-^, *p53*^-/- ^*Atm*^+/-^, and *p53*^-/- ^*Atm*^-/-^). Thus, the significance of haploinsufficiency of the *Atm *null allele in mammary carcinogenesis is obscure at present.

In order to determine the effect of *Atm *heterozygous deficiency on mammary tumourigenesis, we generated a series of *Atm*^+/- ^mice on the background of *p53*^+/- ^mice with a certain predisposition to spontaneous development of mammary carcinomas, and we examined the development of tumours after X-irradiation. Our results indicate a strong enhancement of mammary carcinogenesis in the *Atm *heterozygous deficient mice under the *p53 *heterozygous deficiency.

## Materials and Methods

### Mice

The *p53 *targeted allele generated by Donehower and coworkers [22] was introduced into the BALB/cHeA mouse at The Netherlands Cancer Institute (Amsterdam). The *p53 *heterozygous deficient mice (*p53*^+/-^) were repeatedly backcrossed to BALB/cHeA mice more than 30 times, and maintained at the animal facility of Osaka Prefecture University. The *Atm *targeted mouse (129/SvEv-*Atm*^*tm1Awb*/+ ^mouse) was originally generated in the Jackson Laboratory [23]. The *Atm *heterozygous deficient mice (*Atm*^+/-^) were repeatedly backcrossed more than 10 times to MSM/Ms mice. The BALB/cHeA-*p53*^+/- ^mice were crossed with MSM/Ms-*Atm*^+/- ^mice, and females of the F_1 _progeny ([BALB/cHeA × MSM/Ms]F_1_) with four genotypes (i.e. *p53*^+/- ^*Atm*^+/-^, *p53*^+/- ^*Atm*^+/+^, *p53*^+/+ ^*Atm*^+/- ^and *p53*^+/+ ^*Atm*^+/+^) were used in the experiments. The conditions for breeding were described previously [24].

### X-irradiation

Mice were exposed at 5 weeks of age to X-rays (5 Gy; 260 kV, 12.0 mA, 0.3 mm Cu + 0.5 mm Al filter; 0.5 Gy/min) from an X-ray generator (Radioflex 350; Rigaku Industrial Co., Takatsuki, Japan). All animal experiments were carried out in accordance with the standards relating to the care and management of experimental animals (Japan) and Osaka Prefecture University's guidelines for animal care and use.

### Histopathological examination

Moribund mice were killed by cervical dislocation for autopsy. In cases of thymic lymphoma, the enlarged thymuses were examined as previously described [25]. In tumour-bearing mice the tumours were fixed in 10% buffered formalin, processed histologically, and stained with haematoxylin and eosin. The processed tumour specimens were evaluated by medical and veterinary pathologists using the Annapolis guidelines established by Cardiff and coworkers [26].

### DNA isolation and genotyping

Normal and mammary carcinoma tissues were removed. Isolation of DNA, PCR amplification, electrophoresis of PCR products and assessment of allelic losses were performed according to a procedure described previously [24]. Genotypes for the wild-type and targeted alleles of *p53 *and *Atm *genes were determined by analyzing the PCR products for these alleles. Amplification for the *p53 *alleles was done as described elsewhere [27]. The wild-type and the targeted alleles of the *p53 *gene were amplified by PCR using primers *p53*-4F (5'-CGACCTCCGTTCTCTCTCCTCTCTT-3') and *p53*-6R (5'-AGACGCACAAACCAAAACAAAATTACA-3'), and primers *p53*-NF (5'-GCCTTCTATCGCCTTCTTGACGAGT-3') and *p53*-6R, respectively. Similarly, amplification of the wild-type and the targeted allele of the *Atm *gene were performed by using primers IMR0640F (5'-GCTGCCATACTTGATCCATG-3') and IMR0641R (5'-TCCGAATTTGCAGGAGTTG-3'), and primers IMR0640F and AtmNeo410R (5'-CGGTGGATGTGGAATGTGTG-3'), respectively.

### Statistical analysis

Statistical significance was evaluated for the incidence of mammary carcinoma and number of carcinomas per mouse by χ^2 ^analysis and Mann–Whitney U-test, respectively. Comparison of latency in mammary carcinoma development was examined by unpaired Student's t-test.

## Results and discussion

### Histopathological features of tumours developed in F_1 _mice doubly heterozygous for *p53 *and *Atm *null alleles

**Figure 1:**
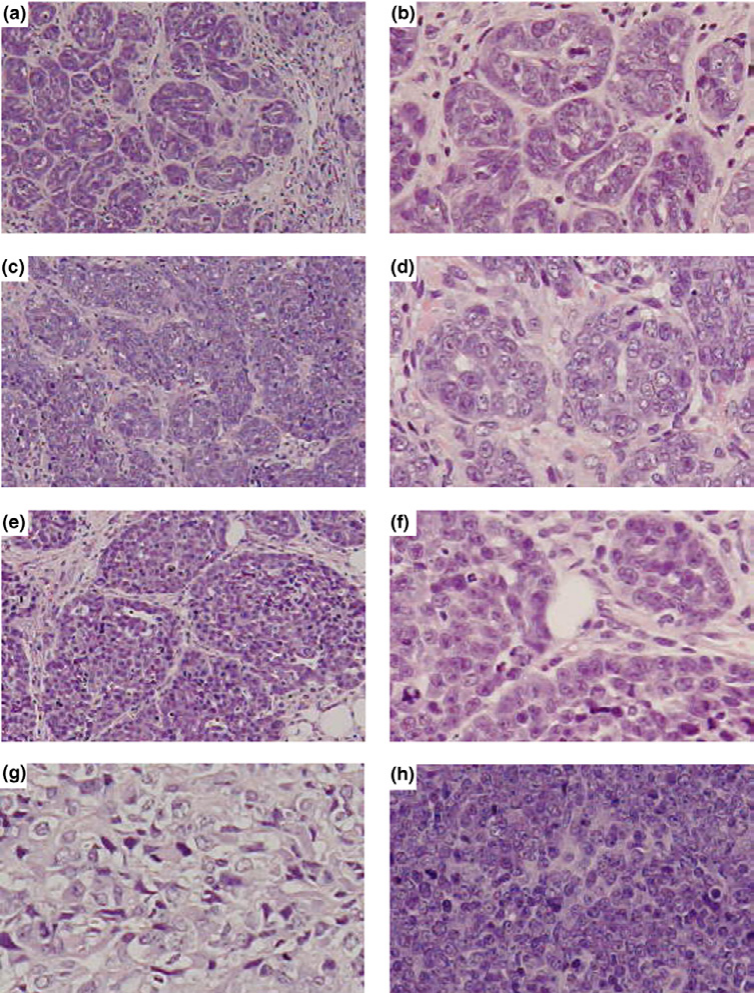
Histopathology of tumours in (BALB/cHeA × MSM/Ms)F_1 _mice with genotypes *p53*^+/- ^*Atm*^+/-^, *p53*^+/- ^*Atm*^+/+ ^or *p53*^+/+ ^*Atm*^+/-^. **(a, b) **Spontaneously developing mammary adenocarcinoma from *p53*^+/- ^*Atm*^+/- ^mouse. **(c, d) **Mammary adenocarcinoma from X-irradiated *p53*^+/- ^*Atm*^+/- ^mouse. **(e, f) **Mammary adenocarcinoma from X-irradiated *p53*^+/- ^*Atm*^+/+ ^mouse. **(g) **Osteosarcoma observed in nonirradiated *p53*^+/- ^*Atm*^+/- ^mouse. **(h) **Thymic lymphoma from *p53*^+/+ ^*Atm*^+/- ^mouse exposed to X-rays. Panels a, c and e: 40×. Panels b, d, f, g and h: 100×.

Mammary carcinomas occurred in BALB/c mice of the *p53*^+/- ^genotype, and tumours of similar macroscopic morphologies were also observed in the (BALB/c × MSM/Ms)F_1_–*p53*^+/- ^mice. The histological features of these tumours in the mammary glands are shown in Fig. 1a,1b,1c,1d,1e,1f, which indicates that they are adenocarcinomas. The histopathology of the tumours in nonirradiated mice (Fig. 1a,1b) exhibited more hyperplastic lesions than did those in irradiated mice (Fig. 1c,1d,1e,1f), but basically there were no marked differences between the two groups. There was also no remarkable difference in histopathological features between *Atm*^+/- ^(Fig. 1c,1d) and *Atm*^+/+ ^mice (Fig. 1e,1f) in the irradiated groups. These tumours formed glands lined by highly pleomorphic cells exhibiting frequent mitosis, and were classified as glandular adenocarcinomas, high grade according to the Annapolis Pathology Classification [26].

Lymphomas (Fig. 1g), mainly thymic lymphomas, were efficiently induced by exposure to X-rays, regardless of *p53 *and *Atm *genotype, although only a few lymphomas were observed in nonirradiated mice. Ovarian carcinomas, osteosarcomas (Fig. 1h) and hepatomas developed spontaneously. Squamous cell carcinomas, basal cell carcinomas, histiocytic sarcomas and granulocytic leukaemias were also observed in irradiated mice.

### Spontaneous tumour development in mice with four genotypes for *p53 *and *Atm*

**Figure 2:**
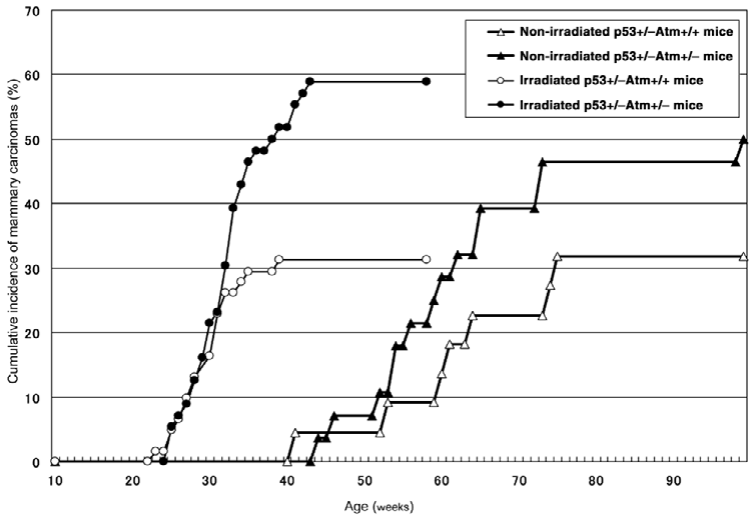
Cumulative incidence of mammary carcinomas in irradiated and nonirradiated (BALB/cHeA × MSM/Ms)F_1 _mice with *p53*^+/- ^*Atm*^+/- ^or *p53*^+/- ^*Atm*^+/+ ^genotype.

Twenty-eight *p53*^+/- ^*Atm*^+/-^, 22 *p53*^+/- ^*Atm*^+/+^, 11 *p53*^+/+ ^*Atm*^+/- ^and 25 *p53*^+/+ ^*Atm*^+/+ ^mice were examined for spontaneous development of tumours until age 26 months (113 weeks). Fourteen out of 28 *p53*^+/- ^*Atm*^+/- ^mice (50%) and seven out of 22 *p53*^+/- ^*Atm*^+/+ ^mice (32%) developed mammary carcinomas during the period of observation (Table 1, Fig. 2). The incidence of mammary carcinomas in *p53*^+/- ^*Atm*^+/- ^mice appeared to be higher than that in *p53*^+/- ^*Atm*^+/+ ^mice, but the incidences did not differ significantly between *Atm*^+/- ^and *Atm*^+/+ ^genotypes (*P *= 0.32, by Fisher's exact probability test). Among the tumours that developed in the *p53*^+/- ^mice, mammary carcinoma were the most common. These mammary carcinomas were mainly observed at 41–77 weeks after birth (Fig. 2). No significant difference in latency in the nonirradiated groups was observed between doubly heterozygous mice and *p53 *singly heterozygous mice (*P *= 0.47, by unpaired Student's t-test). None of 11 *p53*^+/+ ^*Atm*^+/- ^mice and 25 *p53*^+/+ ^*Atm*^+/+ ^mice developed mammary carcinomas. Several lymphomas and a few other tumours developed in the four genotypes (Table 1). Thus, mammary carcinoma development depended strongly on *p53 *heterozygous deficiency in (BALB/c × MSM/Ms)F_1 _mice, and *p53*^+/- ^mice of both *Atm*^+/+ ^and *Atm*^+/- ^genotypes developed mammary carcinoma.

### Enhancement of mammary carcinogenesis in *Atm *heterozygous deficient mice by X-irradiation

To test the effect of haploinsufficiency of the *Atm *gene on mammary carcinogenesis after X-irradiation in *p53*^+/- ^mice, 55 *p53*^+/- ^*Atm*^+/-^, 61 *p53*^+/- ^*Atm*^+/+^, 47 *p53*^+/+ ^*Atm*^+/- ^and 53 *p53*^+/+ ^*Atm*^+/+ ^mice (for a total of 216 mice) were exposed to X-rays (5 Gy) at age 5 weeks. Only one out of 53 *p53*^+/+ ^*Atm*^+/+ ^mice and none of 47 *p53*^+/+ ^*Atm*^+/- ^mice developed mammary carcinoma, indicating that almost all *p53*^+/+ ^mice fail to develop mammary carcinomas, despite X-irradiation and irrespective of *Atm *gene status. In contrast, 32 out of 55 *p53*^+/- ^*Atm*^+/- ^mice (58%) and 19 out of 61 *p53*^+/- ^*Atm*^+/+ ^mice (31%) developed mammary carcinomas (Table 2, Fig. 2). The proportion of mice developing mammary carcinomas in the *p53*^+/- ^*Atm*^+/- ^group was significantly greater than that in the *p53*^+/- ^*Atm*^+/+ ^group (*P *= 0.0034, by χ^2 ^test). A total of 52 mammary carcinomas developed in 55 *p53*^+/- ^*Atm*^+/- ^mice (average number of mammary carcinomas/mouse = 0.95), whereas 28 mammary carcinoma developed in 61 *p53*^+/- ^*Atm*^+/+ ^mice (average number of mammary carcinomas/mouse = 0.46; Table 2). The average number of mammary carcinomas per mouse in the *p53*^+/- ^*Atm*^+/- ^group was significantly greater than that in *p53*^+/- ^*Atm*^+/+ ^mice (*P *= 0.0052, by Mann–Whitney U-test). Thus, *Atm *heterozygous deficiency enhanced development of mammary carcinoma in irradiated *p53 *heterozygous knockout mice. Spring and coworkers [14] observed no tumours in *Atm *knockout (*Atm*^+/-^) heterozygous mice. Mice bearing a knockout allele of *Atm *and humans carrying a mutant allele of truncated type in the *ATM *gene have been shown not to have obviously elevated susceptibility to mammary carcinogenesis. Our findings show that heterozygosity for a null knockout allele of *Atm *enhances mammary carcinogenesis under *p53*^+/- ^status, although the *Atm *mutation is not a dominant-negative type. Heterozygous deficiency of *p53 *might make clear the effect on mammary carcinogenesis of haploinsufficiency in the *Atm *gene.

These mammary carcinomas were observed significantly earlier (at 18–38 weeks after irradiation; i.e. 23–43 weeks of age) than in the nonirradiated group (age 41–75 weeks; Fig. 2). In particular, mammary carcinomas frequently developed 23–28 weeks after irradiation. The mean (± standard deviation) latency periods were 32.6 ± 4.8 and 29.8 ± 3.6 weeks in *p53*^+/- ^*Atm*^+/- ^and *p53*^+/- ^*Atm*^+/+ ^mice, respectively. Thus, X-irradiation at 5 Gy at age 5 weeks considerably shortened the latency period of mammary carcinoma development in these two groups with different genotypes. As shown in Tables 1 and 2, the incidences of mammary carcinoma in *p53*^+/- ^*Atm*^+/- ^mice were 58% (32 out of 55) and 50% (14 out of 28) in irradiated and nonirradiated groups, respectively; in *p53*^+/- ^*Atm*^+/+ ^mice the incidence in the irradiated group was 31% (19 out of 61) and that in the nonirradiated group was 32% (7 out of 22). The incidence of mammary carcinoma for each genotype was similar between irradiated and non-irradiated groups. Thus, irradiation may not elevate the incidence of the tumours.

Altogether, irradiation markedly hastened mammary carcinoma development in the *p53*^+/- ^mice, in which mammary carcinomas developed spontaneously. Furthermore, irradiation also induced lymphomas, mainly thymic lymphomas, in all four genotypes of mice. The incidence of the lymphomas did not differ significantly among the four groups, with different genotypes for *p53 *and *Atm *genes. A high incidence of thymic lymphoma was observed in previous studies performed using *p53 *heterozygous deficient F_1 _mice [17,28]. In the present study an extremely high incidence of tumours, most of which were mammary carcinomas and thymic lymphomas, was observed in irradiated *p53*^+/- ^*Atm*^+/- ^mice.

### Status of wild-type alleles of *p53 *and *Atm *in mammary carcinoma

The wild-type alleles of the *p53 *and *Atm *genes were examined in mammary carcinoma tissue from heterozygous mice. The wild-type *p53 *allele was lost in 25 (96%) out of 26 mammary carcinomas from irradiated *p53*^+/- ^mice and in all of 15 mammary carcinomas from nonirradiated *p53*^+/- ^mice, regardless of *Atm *gene status. Only one mammary carcinoma in irradiated *p53*^+/+ ^*Atm*^+/+ ^mice (Table 2) was found to retain the *p53 *wild-type allele. On the other hand, wild-type *Atm *allele was preserved in all of 17 mammary carcinomas from irradiated *Atm*^+/- ^mice and in all of 10 mammary carcinomas from irradiated *Atm*^+/+ ^mice, regardless of *p53 *gene status. Wild-type *Atm *allele was also retained in nine out of 10 mammary carcinomas from nonirradiated *Atm*^+/- ^mice and in all of five mammary carcinomas from nonirradiated *Atm*^+/+ ^mice. These results suggest that the homozygous loss of the *p53 *allele was a necessary condition for the development of mammary carcinomas, whereas the *Atm *null allele exhibited haploinsufficiency.

Haploinsufficiency for tumour development was reported for the *Nbn *knockout mice, the mouse homologue of *NBS1*. Heterozygosity for the *Nbn *knockout allele rendered the mice susceptible to tumour development, yet the *Nbn *wild-type allele was fully retained in all 12 tumours examined [29]. *p27 *heterozygotes are also predisposed to tumours in multiple tissues when challenged with irradiation or a chemical carcinogen, and in the developed tumours the remaining wild-type allele is neither mutated nor silenced, indicating that *p27 *is haploinsufficient for tumour suppression [30]. In that study, heterozygous *Atm *knockout enhanced mammary carcinoma development in *p53*-heterozygous deficient mice, but the effect of *Atm *deficiency was not as profound. A previous study showed that heterozygosity for the *Atm *knockout allele did not enhance tumour development but that dominant-negative type missense mutations in the *Atm *gene did [14]. In humans, heterozygosity of the truncation-type mutations, which represent the majority of *Atm *mutations that occur in humans, had no effect on carcinogenesis, suggesting that enhancement in tumourigenesis depends strongly on mutation type. However, in the present study we demonstrated that haploinsufficiency does occur for the *Atm *null allele in combination with heterozygous deficiency in the *p53 *gene. The *p53 *heterozygous deficient BALB/c mice, which developed mammary carcinomas early and efficiently, may represent a useful model for the study of effects of genes other than *Atm *on mammary carcinogenesis. F_1 _mice between different subspecies may also provide an experimental system for precise genome-wide allelotype analysis of genes that cooperate with *p53*.

## Conclusion

Tumourigenesis is strongly enhanced in mice with homogeneous deficiency in the *p53 *or *Atm *gene. In the present study we tested the effect of haploinsufficiency of the *Atm *gene on mammary tumourigenesis after X-irradiation in *p53*^+/- ^mice of the BALB/cHeA × MSM/Ms background. Singly heterozygous *p53*^+/- ^mice X-irradiated (5 Gy) at age 5 weeks developed mammary carcinomas at around 25 weeks of age, and the final incidence of mammary carcinoma at 39 weeks was 31% (19 out of 61). Introduction of the heterozygous *Atm *alleles into the background of the *p53*^+/- ^genotype significantly increased the incidence of mammary carcinomas to 58% (32 out of 55) and increased the average number of mammary carcinomas per mouse. However, it apparently did not change the latency of mammary carcinoma development. In nonirradiated mice, introduction of the *Atm*^+/- ^allele into *p53*^+/- ^mice also tended to increase spontaneous incidence of mammary carcinoma. In contrast, almost none of the *p53*^+/+ ^mice developed mammary carcinoma, regardless of the *Atm *gene status and whether mice were subjected to irradiation. In almost all of the spontaneous and radiation-induced mammary carcinomas, the wild-type *p53 *allele was found to be lost whereas the wild-type *Atm *allele was retained, suggesting haploinsufficiency of the latter gene in mammary carcinoma development. Thus, doubly heterozygous mice represent a useful model system with which to analyze the interaction of heterozygous genotypes for *p53*, *Atm *and other genes and their effect on mammary carcinogenesis.

## Abbreviations

PCR = polymerase chain reaction.

## Competing interests

The author(s) declare that they have no competing interests.

## Authors' contributions

SU carried out design of the study, observed mammary carcinoma development and other diseases, and drafted a manuscript. KF prepared tissue specimens and conducted histopathological examinations (veterinarian). SI carried out DNA isolation and genotyping of mice. NM carried out X-irradiation of mice and statistical analysis. MT performed DNA isolation and genotyping of mice. DH carried out production of heterozygous deficient mice. CS participated in designing the study and discussion of data on mammary carcinoma development. SH contributed to histopathological examination. SI conducted histopathological examinations (medical doctor). ON participated in discussion of data and contributed to preparation of the final manuscript. MO carried out design of the study and X-irradiation, and wrote the final manuscript. All authors read and approved the final manuscript.

## Figures and Tables

**Table 1:** Incidence of tumours developing spontaneously in (BALB/cHeA × MSM/Ms)F_1 _female mice that were heterozygously deficient for *p53 *and/or *Atm *genes

Genotypes	*p53*^+/-^		*p*53^+/+^	
	
	*Atm*^+/- ^(*n *= 28)	*Atm*^+/+ ^(*n *= 22)	*Atm*^+/- ^(*n *= 11)	*Atm*^+/+ ^(*n *= 25)
Mammary carcinoma	14 (50%)^a^	7 (32%)	0 (0%)	0 (0%)
Lymphoma	3 (11%)	3 (14%)	2 (18%)	4 (16%)
Ovarian carcinoma	1 (4%)	1 (5%)	0 (0%)	1 (4%)
Osteosarcoma	1 (4%)	0(0%)	0 (0%)	0 (0%)
Hepatoma	1 (4%)	0(0%)	0 (0%)	0 (0%)
Number of mammary carcinomas/mouse^b^	14/28 (0.50)	7/22 (0.32)	0/11 (0.00)	0/25 (0.00)

^a^Number of mice with tumours. ^b^Total number of mammary carconomas developing in mice with given genotypes/number of mice examined; numbers in parentheses are average numbers of tumours per mouse.

**Table 2:** Incidence of tumours in irradiated (BALB/cHeA × MSM/Ms) F_1 _female mice that were heterozygously deficient for *p53 *and/or *Atm *genes

Genotypes	*p*53^+/-^		*p53*^+/+^	
	
	*Atm*^+/- ^(*n *= 55)	*Atm*^+/+ ^(*n *= 61)	*Atm*^+/- ^(*n *= 47)	*Atm*^+/+ ^(*n *= 53)
Mammary carcinoma	32 (58%)^a^*	19 (31%)	0 (0%)	1 (2%)
Lymphoma	24 (44%)^b^	29 (48%)	26 (55%)	22 (42%)
Squamous cell carcinoma	1 (2%)	1 (2%)	0 (0%)	0 (0%)
Histiocytic sarcoma	1 (2%)^c^	0 (0%)	0 (0%)	0 (0%)
Basal cell carcinoma	0 (0%)	1 (2%)	0 (0%)	0 (0%)
Granulocytic leukaemia	0 (0%)	1 (2%)	0 (0%)	0 (0%)
Ovarian carcinoma	0 (0%)	1 (2%)	0 (0%)	0 (0%)
Nonthymic lymphoma (NOS)	0 (0%)	1 (2%)	3 (6%)	0 (0%)
Solid tumour (NOS)	1 (2%)^d^	1 (2%)^e^	1 (2%)	1 (2%)
Number of mammary carcinomas/mouse^e^	52/55 (0.95)**	28/61 (0.46)	0/47 (0.00)	1/53 (0.02)

^a^Number of mice with tumours; percentage in parenthesis is the proportion of mice developing mammary carcinoma. ^b^Three animals developed both lymphomas and mammary carcinoma. ^c^One animal developed both histiocytic sarcoma and mammary carcinoma. ^d^Tumour in abdomen. ^e^Total number of mammary carcinomas developing in mice with given genotypes/number of mice examined; numbers in parentheses are average numbers of tumours per mouse. *The proportion of mice developing tumours is significantly greater than that in *p53*^+/- ^*Atm*^+/+ ^mice (*P *= 0.0034 by c^2 ^test). **The average number of tumours/mouse is significantly greater than that in *p*53^+/- ^*Atm*^+/+ ^mice (*P *= 0.0052 by Mann–Whitney U-test). NOS, not otherwise specified.
